# Murray law-based quantitative flow ratio for optimizing revascularization in anterior ST-elevation myocardial infarction due to spontaneous coronary artery dissection: a case report

**DOI:** 10.1093/ehjcr/ytaf524

**Published:** 2025-10-10

**Authors:** Alberto Polimeni, Giovanni Martino, Simone Biscaglia, Ciro Indolfi, Antonio Curcio

**Affiliations:** Department of Pharmacy, Health and Nutritional Sciences, University of Calabria, Rende 87035, Italy; Division of Cardiology, Annunziata Hospital, Cosenza 87100, Italy; Department of Medical and Surgical Sciences, Magna Graecia University, Catanzaro 88100, Italy; Cardiovascular Institute, Azienda Ospedaliero-Universitaria di Ferrara, Cona 44124, Italy; Department of Pharmacy, Health and Nutritional Sciences, University of Calabria, Rende 87035, Italy; Department of Pharmacy, Health and Nutritional Sciences, University of Calabria, Rende 87035, Italy; Division of Cardiology, Annunziata Hospital, Cosenza 87100, Italy

**Keywords:** Case report, Acute coronary syndrome (ACS), Spontaneous coronary artery dissection (SCAD), Murray law-based quantitative flow ratio (μQFR)

## Abstract

**Background:**

Spontaneous coronary artery dissection (SCAD) is a rare, non-atherosclerotic cause of acute coronary syndrome (ACS) characterized by compression of the coronary true lumen by a blood-filled false lumen. Conservative management is recommended for most cases, with percutaneous coronary intervention (PCI) reserved for patients with significant ischaemia, reduced coronary blood flow, or haemodynamic instability. Percutaneous coronary intervention in SCAD is challenging, with a high complication rate, and the optimal approach remains debated.

**Case summary:**

We describe a 43-year-old woman presenting with acute retrosternal chest pain and ST-segment elevation consistent with an anterior ST-elevation myocardial infarction. The patient medical history included hypertension, smoking, recent hormonal therapy, and prednisone use. Emergent coronary angiography revealed a type IIb SCAD in the mid-left anterior descending artery with vessel occlusion. Plain balloon angioplasty successfully restored good antegrade flow (TIMI 3), deferring the decision of stent implantation on follow-up coronary angiography. Murray law-based quantitative flow ratio (μQFR) revealed a haemodynamically non-significant lesion at 10 days of follow-up. The patient remained stable, demonstrating recovery of myocardial function and no recurrent symptoms at 12-months of follow-up.

**Discussion:**

Murray law-based quantitative flow ratio offers a non-invasive tool to guide revascularization in SCAD patients. While promising, evidence for μQFR in SCAD is lacking, highlighting the need for further studies to validate its utility in this context.

Learning points

- Spontaneous coronary artery dissection (SCAD) can be life-threatening when presenting as acute coronary syndrome, especially in ST-elevation myocardial infarction cases. While conservative treatment is preferred when possible, invasive management may sometimes be required.
- Percutaneous coronary intervention in SCAD is technically challenging and carries a high complication risk. Murray law-based quantitative flow ratio may be a promising tool to guide revascularization decisions in these patients.

## Introduction

Spontaneous coronary artery dissection (SCAD) is a rare, non-atherosclerotic cause of acute coronary syndrome, characterized by compression of the coronary true lumen by a blood-filled false lumen. This false lumen can develop through two mechanisms: an intimal tear (‘inside-out’) or an intramural haematoma due to vasa vasorum haemorrhage (‘outside-in’).^1^ Spontaneous coronary artery dissection manifests across a wide clinical spectrum, from ACS to severe cardiogenic shock caused by extensive myocardial ischaemia. The most recent European Society of Cardiology (ESC) guidelines recommend conservative medical management for SCAD whenever feasible, as it is effective in most cases. Percutaneous coronary intervention (PCI) is reserved (Class I, Level of Evidence C) only in cases of SCAD associated with ongoing myocardial ischaemia, significant myocardial territory at risk, or reduced antegrade coronary blood flow.^2^ However, PCI in SCAD is associated with a high complication rate, occurring in approximately 40% of cases, with serious complications reported in 13%.^3^ Due to the rarity of SCAD, the optimal treatment approach is still debated. Furthermore, in cases where invasive management is pursued, predictors of PCI success in this setting represent a significant gap in current evidence.

In this report, we present a clinical case of SCAD managed using Murray law-based quantitative flow ratio (μQFR), an angiography-derived physiological index. Murray law-based quantitative flow ratio was chosen for its specific advantages in this clinical context. Compared to standard QFR, which requires two angiographic projections and complex 3D vessel reconstruction, μQFR relies on a single angiographic view and applies fractal geometry based on Murray’s law to estimate coronary flow. This makes the method faster, less dependent on imaging quality or operator expertise, and particularly suitable for emergency or technically challenging settings. Importantly, μQFR avoids the need for intracoronary instrumentation, which is especially relevant in SCAD, where manipulation of pressure wires may carry the risk of entering the false lumen and worsening the dissection.

## Summary figure

**Figure:**
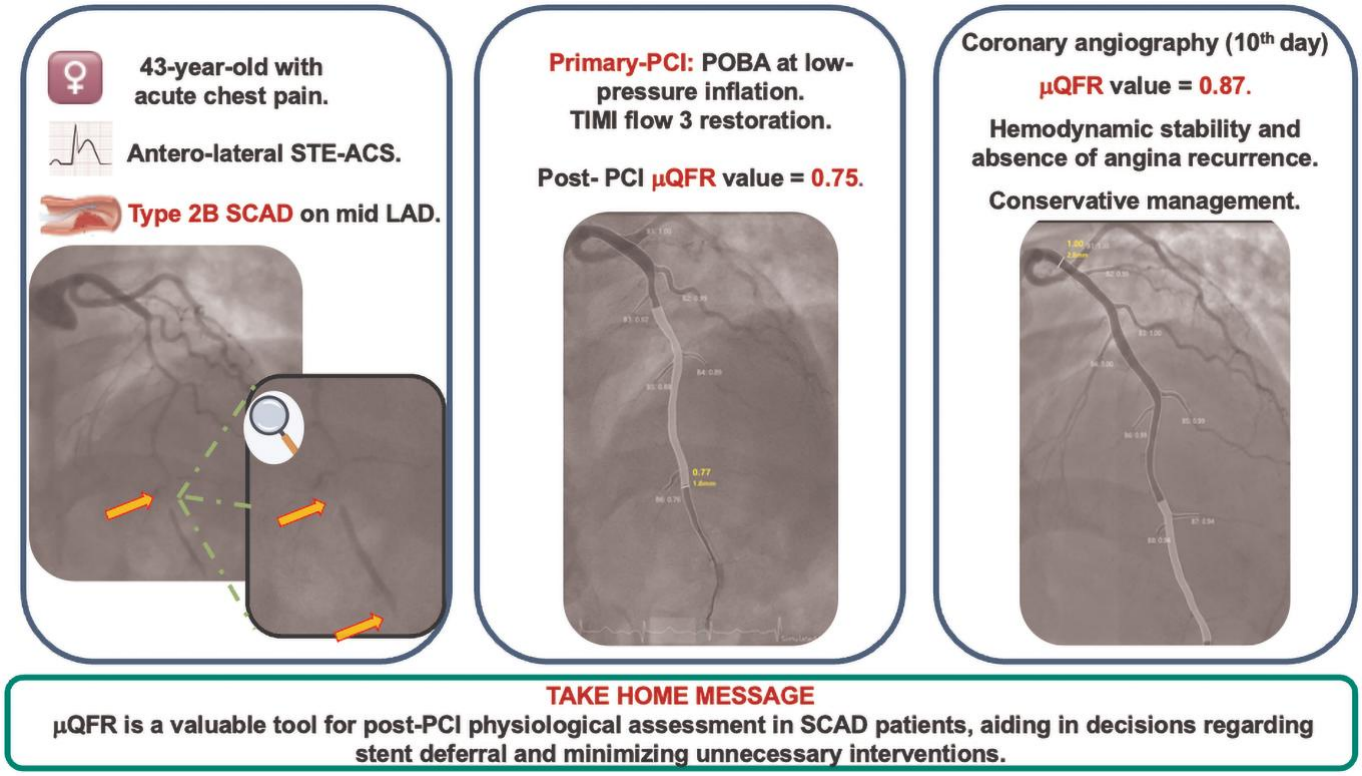


## Case presentation

**Figure 1:**
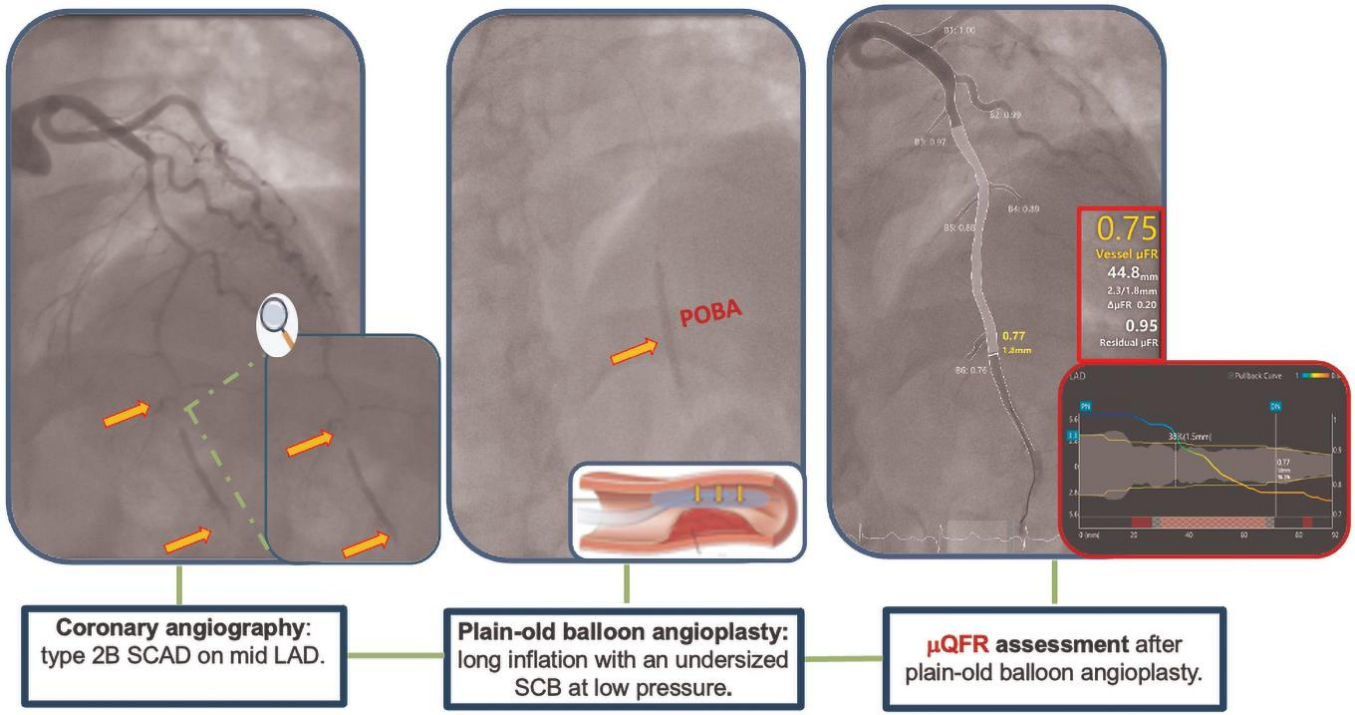
Coronary angiography and Murray law-based quantitative flow ratio assessment after plain-old balloon angioplasty in a type 2B spontaneous coronary artery dissection of the left anterior descending artery.

We present the case of a 43-year-old woman who was admitted to the emergency department with acute retrosternal chest pain that began after an emotional distress. The medical history was notable for a smoking habit and arterial hypertension managed pharmacologically with an angiotensin receptor blocker. The patient had also undergone hormonal therapy with chorionic gonadotropin and clomiphene citrate for fertility purposes up to 8 months prior to this presentation. Additionally, she had recently undergone a uterine polypectomy and was receiving prednisone at a daily dose of 25 mg following the procedure. On admission, the patient was afebrile, with a blood pressure of 190/95 mmHg and a heart rate of 105 b.p.m. A 12-lead electrocardiogram revealed ST-segment elevation in the anterolateral leads, consistent with an acute ST-elevation myocardial infarction (STEMI). In the emergency department, she was treated with intravenous acetylsalicylic acid (ASA), unfractionated heparin (UFH), sodium nitroglycerin, and prasugrel with a loading dose. The patient was then promptly transferred to the cardiac catheterization laboratory for emergent coronary angiography (CAG). Coronary angiography revealed a type 2B SCAD involving the mid-left anterior descending coronary artery, resulting in vessel occlusion (TIMI flow grade 0–1). Since the vessel was occluded, performing a μQFR assessment before PCI was not appropriate nor technically feasible, as adequate contrast flow is required to allow for a reliable analysis. Given the persistency of symptoms and the antegrade flow-limiting nature of the SCAD lesion, we opted for an invasive approach. Using a non-polymeric, non-hydrophilic coronary guidewire to minimize the risk of entering the false lumen, we performed plain old balloon angioplasty (POBA) with an undersized semi-compliant balloon (2.0 × 20 mm) inflated at low atmospheres for 30 s and gradually deflated, leading to successfully restored antegrade distal flow in the affected vessel. As optical coherence tomography (OCT) was not available in our laboratory at the time, we assessed the haemodynamic impact of the dissection non-invasively using μQFR, which revealed a value of 0.75 (post-POBA μQFR). This finding indicated a haemodynamically significant lesion characterized by a diffuse pattern of disease (*Figure 1*).

**Figure 2:**
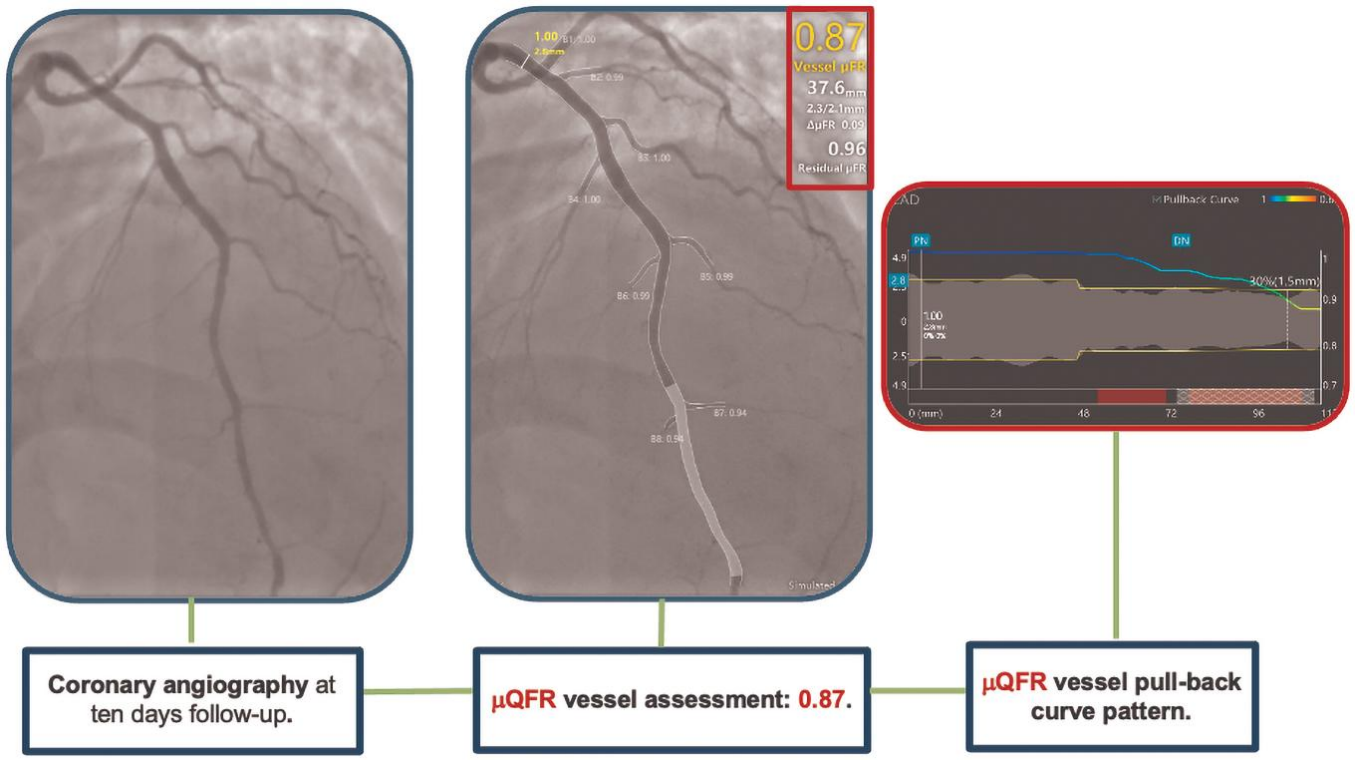
Coronary angiography at 10 days of follow-up and Murray law-based quantitative flow ratio assessment of the left anterior descending artery.

Our primary goals were to restore coronary flow, ensure adequate myocardial perfusion, preserve cardiac function, and resolve the patient’s symptoms, including ST-segment elevation. Based on these considerations, we opted not to stent the dissection, as we trusted that our intervention during the index procedure would lead to an improvement in flow and consequently in the μQFR during follow-up. We scheduled a new coronary angiography 10 days later to reassess the situation and determine the most appropriate treatment strategy, whether conservative or interventional. The patient was subsequently transferred to the intensive coronary care unit (ICU) for targeted medical management. Blood tests revealed no significant abnormalities except for a markedly elevated high-sensitivity troponin I (hs-TnI) level of 64 326.6 ng/L (normal range < 14 ng/L). Transthoracic echocardiography (TTE) demonstrated apical hypokinesia, leading to a reduced left ventricular ejection fraction of 45%, with preserved right ventricular systolic function. The patient experienced no recurrent episodes of angina and remained haemodynamically stable during the following days. A statin and a more tailored antihypertensive regimen were prescribed. Additionally, 24 h after the last prasugrel loading dose, prasugrel was switched to clopidogrel on top of ASA. A follow-up coronary angiography was performed 10 days later, confirming a favourable angiographic result and an improved μQFR measurement of 0.87 (*Figure 2*).

**Figure 3:**
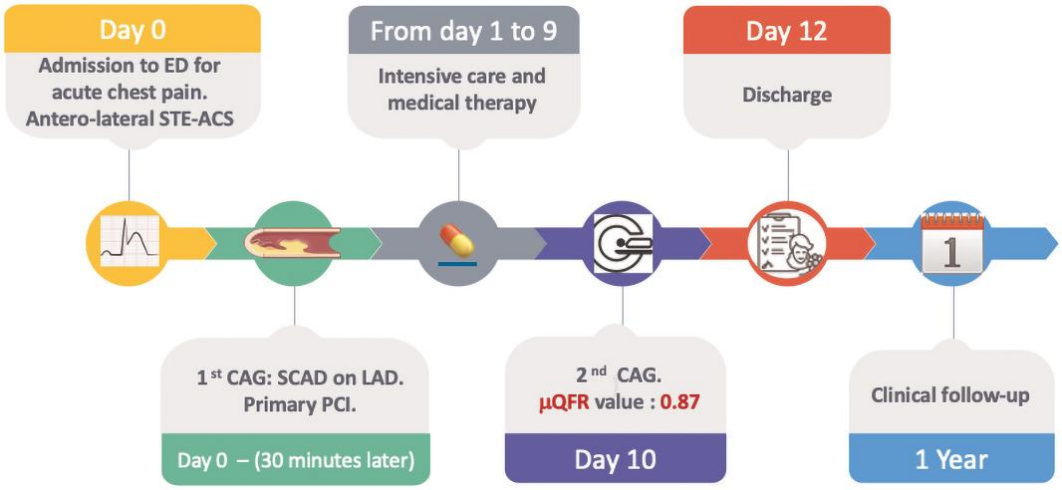
Timeline of events.

Given the improvement in μQFR value, the patient’s haemodynamic stability, and the absence of recurrent anginal episodes, a conservative management strategy was adopted. The patient was discharged home the 12th day of hospitalization, with not experienced major cardiac arrhythmias. At 12 months of follow-up, the patient reported no symptoms, good arterial blood pressure control, and normal levels of serial biomarkers (*Figure 3*).

## Discussion

Spontaneous coronary artery dissection is estimated to account for 1%–4% of acute coronary syndrome cases overall and up to 35% of myocardial infarctions (MIs) in women aged ≤50 years.^4^ Spontaneous coronary artery dissection typically affects young to middle-aged women with minimal to low cardiovascular (CV) risk factors. Clinically, SCAD usually presents as ACS, characterized by anginal chest pain and elevated cardiac enzyme levels. Approximately 36.8% of cases manifest as STEMI, while 48.5% present as NSTEMI.^5^ Once SCAD is suspected, coronary angiography is essential and serves as the primary diagnostic tool in most cases.^4^ The primary goals of acute SCAD management are to preserve minimal coronary flow, maintain myocardial perfusion and cardiac function, alleviate symptoms, and prevent immediate complications. Percutaneous coronary intervention outcomes in SCAD are less predictable due to higher rates of complications, including iatrogenic haematoma propagation, dissection extension, abrupt vessel occlusion, or stent malposition. Consequently, interventionalists often adopt a conservative approach whenever feasible. Revascularization is reserved for a minority of patients with severe clinical presentations, such as ongoing ischaemia, haemodynamic instability, or high-risk anatomical features (e.g. left main dissection or severe proximal coronary occlusion).^6^ For such cases, strategies include minimal plain balloon angioplasty to restore coronary flow, targeted stenting to seal dissection edges, and/or the use of extended stent lengths to prevent haematoma propagation. A minimalistic approach is typically employed following revascularization when achievable.^7^

Various procedural strategies for PCI in SCAD lesions have been proposed, including POBA, cutting balloons (CB),^8^ and bioresorbable vascular scaffolds^9^ as alternatives to drug-eluting stents (DES) for preserving coronary vessel physiology. Additionally, advanced coronary imaging techniques have been employed to mitigate PCI-related complications.^10^ However, a standardized definition of procedural success in this specific context remains uncertain.

In this setting, μQFR offers a valuable approach for assessing PCI result in SCAD. Murray law-based quantitative flow ratio estimates fractional flow reserve (FFR) by analysing a single angiographic projection, adjusting the reference vessel diameter, and outgoing flow through side branches according to fractal geometry principles.^11^ This approach eliminates the need for pressure wires or adenosine infusion, offering a less invasive and safer alternative. Importantly, µQFR provides a quantitative assessment of myocardial perfusion restoration, which is particularly relevant in SCAD cases where visual evaluation alone may be insufficient and where the additional requirement to wire the vessel with a pressure wire, as in standard FFR, could enter the false lumen and pose additional significant risks.

The use of FFR in the management of atherosclerotic lesions following POBA has been historically advocated to better predict clinical outcomes.^12^ Recent studies have demonstrated that post-POBA FFR values greater of 0.85^13^ and even 0.75^14^ are associated with a lower likelihood of restenosis and other adverse outcomes. This highlights the significance of suboptimal post-PCI FFR in predicting long-term outcomes. However, no invasive tool is currently validated for physiological assessment in the setting of SCAD. More importantly, this case represents, to the best of our knowledge, the first description of μQFR application in SCAD. While we cannot retrospectively determine whether wire-based physiology or OCT evaluation would have led to the same clinical decision, μQFR proved to be a valuable tool to predict PCI success, aligning with the physiological assessment principles used in conventional invasive approaches.

Importantly, SCAD represents a pathophysiological entity distinct from atherosclerotic disease. When coronary flow is preserved, the most conservative management possible is generally preferred, as SCAD lesions often heal spontaneously over time. In our case, given the preserved TIMI 3 flow and good μQFR value, a conservative approach with POBA was deemed appropriate. If the flow had been impaired, we would have considered alternative strategies—such as cutting balloons to evacuate the intramural haematoma or DES to ensure vessel patency—based on the specific angiographic findings and clinical stability. Ultimately, the guiding principle in SCAD remains to minimize intervention whenever possible.

## Lead author biography


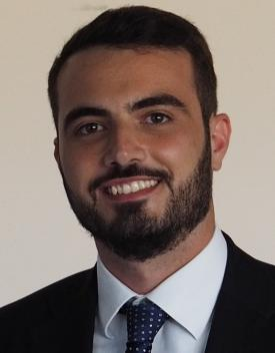


Alberto Polimeni is an assistant professor of Cardiovascular Diseases at the University of Calabria (UNICAL). He achieved a medical degree, a board in cardiology (both with honours), a Ph.D. in Biomarkers of Chronic and Complex Diseases, and a master’s in Biostatistics. He completed a research fellowship at the University Medical Center of Mainz, Germany, and has extensive clinical experience in interventional cardiology. His research focuses on artificial intelligence, robotics, coronary physiology, and structural heart diseases. He serves as an editor and reviewer and has received numerous awards, including being named a Fellow of the European Society of Cardiology (ESC).

## Data Availability

The data supporting the findings of this case report are available from the corresponding author upon reasonable request, subject to privacy and ethical considerations.

## References

[1] Hayes SN, Kim ESH, Saw J, Adlam D, Arslanian-Engoren C, Economy KE, et al Spontaneous coronary artery dissection: JACC state-of-the-art review. J Am Coll Cardiol 2020;76:961–984. 32819471 10.1016/j.jacc.2020.05.084

[2] Byrne RA, Neumann FJ, Akin I, Baumbach A, Baumbach H, Delgado V, et al 2023 ESC guidelines for the management of acute coronary syndromes. Eur Heart J 2023;44:3720–3826. 37622654 10.1093/eurheartj/ehad191

[3] Kotecha D, Singh A, Agostoni P, Agewall S, Andreotti F, Beltrame JF, et al Risks and benefits of percutaneous coronary intervention in spontaneous coronary artery dissection. Heart 2021;107:1398–1406. 34006503 10.1136/heartjnl-2020-318914 PMC8372386

[4] Adlam D, Alfonso F, Maas A, Vrints C. European Society of Cardiology, acute cardiovascular care association, SCAD study group: a position paper on spontaneous coronary artery dissection. Eur Heart J 2018;39:3353–3368. 29481627 10.1093/eurheartj/ehy080 PMC6148526

[5] Petrović M, Baracchini C, Venneri L, De Caterina R. Management and outcomes of spontaneous coronary artery dissection: a systematic review of the literature. Front Cardiovasc Med 2024;11:1–11. 10.3389/fcvm.2024.1276521 PMC10829101 38298759

[6] Tweet MS, Eleid MF, Best PJ, Lennon RJ, Lerman A, Rihal CS, et al Spontaneous coronary artery dissection: revascularization versus conservative therapy. Circ Cardiovasc Interv 2014;7:777–786. 25406203 10.1161/CIRCINTERVENTIONS.114.001659

[7] Morena A, Giannini F, Chiarito M, Regazzoli D, Stefanini GG. Advances in the management of spontaneous coronary artery dissection (SCAD): a comprehensive review. Rev Cardiovasc Med 2024;25:345. 39355597 10.31083/j.rcm2509345 PMC11440404

[8] Yumoto K, Sasaki H, Aoki H, Kato K. Successful treatment of spontaneous coronary artery dissection with cutting balloon angioplasty as evaluated with optical coherence tomography. JACC Cardiovasc Interv 2014;7:817–819. 24954570 10.1016/j.jcin.2013.10.027

[9] Macaya F, Tzoumas A, Salinas P, Gonzalo N, Jiménez-Quevedo P, Fernández-Ortiz A, et al Bioresorbable scaffolds to treat spontaneous coronary artery dissection. Circ Cardiovasc Interv 2016;9:1–4. 10.1161/circinterventions.115.003133 27047996

[10] Macaya F, Tzoumas A, García-García HM, Jiménez-Quevedo P, Gonzalo N, Salinas P, et al Feasibility and safety of intracoronary imaging for diagnosing spontaneous coronary artery dissection. JACC Cardiovasc Imaging 2019;12:763–764. 30553667 10.1016/j.jcmg.2018.09.023

[11] Ding D, Xu B, Matsumura M, Lee JM, Hoshino M, Habara M, et al Quantitative flow ratio based on Murray fractal law: accuracy of single versus two angiographic views. J Soc Cardiovasc Angiogr Interv 2022;1:100399. 39131462 10.1016/j.jscai.2022.100399 PMC11307523

[12] Bech GJW, De Bruyne B, Pijls NH, de Valk V, Hoorntje JC, Koolen JJ, et al Usefulness of fractional flow reserve to predict clinical outcome after balloon angioplasty. Circulation 1999;99:883–888. 10027810 10.1161/01.cir.99.7.883

[13] Shin ES, Ann SH, Singh GB, Lim KH, Kim YJ, Kim MC, et al Fractional flow reserve-guided paclitaxel-coated balloon treatment for de novo coronary lesions. Catheter Cardiovasc Interv 2016;88:193–200. 26423017 10.1002/ccd.26257

[14] Her AY, Kim YH, Park KW, Kim Y, Bae JH, Cho YH, et al Paclitaxel-coated balloon treatment for functionally nonsignificant residual coronary lesions after balloon angioplasty. Int J Cardiovasc Imaging 2018;34:1339–1347. 29696453 10.1007/s10554-018-1351-z

